# The Effect of Antioxidant Added to Preservation Solution on the Protection of Kidneys before Transplantation

**DOI:** 10.3390/ijms23063141

**Published:** 2022-03-15

**Authors:** Aneta Ostróżka-Cieślik

**Affiliations:** Department of Pharmaceutical Technology, Faculty of Pharmaceutical Sciences in Sosnowiec, Medical University of Silesia, Kasztanowa 3, 41-200 Sosnowiec, Poland; aostrozka@sum.edu.pl

**Keywords:** renal transplantation, antioxidants, organ preservation solution

## Abstract

Ischemia–reperfusion injury is a key clinical problem of transplantology. Current achievements in optimizing organ rinse solutions and storage techniques have significantly influenced the degree of graft damage and its survival after transplantation. In recent years, intensive research has been carried out to maintain the viability of tissues and organs outside the integral environment of the body. Innovative solutions for improving the biochemical functions of the stored organ have been developed. The article discusses directions for modifying preservation solutions with antioxidants. Clinical and experimental studies aimed at optimizing these fluids, as well as perfusion and organ preservation techniques, are presented.

## 1. Introduction

Transplantation is now considered an alternative treatment method, allowing for the extension of life of patients with end-stage organ failure and their return to normal functioning. It is often a life-saving method. However, the number of people waiting for a transplant increases disproportionately in relation to the performed transplants. Recently, research has been conducted on the use of marginal donor organs. Therefore, it is important to improve transplantation techniques, including the development of effective methods for organ storage and optimization of solutions intended for graft perfusion and preservation.

## 2. Ischemia-Induced Organ Damage

Maintaining vital functions of the organ outside the donor’s organism is a complex process. Organ ischemia may induce irreversible changes leading to acute rejection, primary or delayed graft function, or initial poor graft function. Damage can be generated in three stages: during disorders of homeostasis and distribution of blood flow through the organ in the donor’s body, during warm (WIT) and cold (CIT) ischemia time, and during reperfusion. The warm ischemia time is the period between the interruption of the blood supply to the organ and the beginning of cold storage. Current in situ techniques of organ cooling practically enable the elimination of the warm ischemia time. However, warm ischemia may occur after cardiac arrest prior to organ procurement. Cold ischemia, in turn, is associated with the period of organ storage by simple hypothermia or constant perfusion [1]. The extent of damage is related to the time that elapses until the organ reperfusion—that is, restoration of circulation. 

During ischemia, cells are deprived of oxygen, and anaerobic transitions are activated, which leads to the accumulation of toxic transformation products. Currently, the aim is to quickly cool the organ after procurement, rinse the blood off the vascular system, and fill the vascular bed with an organ preservation solution [1,2,3]. 

During ischemia, mitochondria are damaged. Their swelling and a change in mitochondrial permeability transition are observed. Stopping aerobic respiration leads to the inhibition of the oxidative phosphorylation process, the exhaustion of high-energy adenosine triphosphate reserves (ATP), and a decrease in the activity of active transport systems and cell membrane potential. The electrolyte composition of the intracellular fluid changes. The sodium–potassium pump becomes inefficient, which means that potassium leaves the cell and sodium enters it [4]. Phosphates (HPO_4_^2−^) are released into the extracellular fluid. The penetration of sodium and chlorine into the cell increases its osmolarity, and as a consequence, leads to the inflow of water and cell swelling [1,5]. The concentration of Ca^2+^ ions increases within the cell. Cell-damaging enzymes are induced, i.e., phospholipases, lysosomal hydrolases, endonucleases, proteases, ATPases [6,7]. Transition to the anaerobic pathway results in the retention of H^+^ ions, lactates, and CO_2_ in the cell and causes its acidification. Lowering the pH in cytosol causes instability of lysosomes, blocks the activity of lysosomal hydrolases, and changes mitochondrial properties. Cytochrome c is released into the cytoplasm, which in turn can lead to cell apoptosis or necrosis [4,8,9]. Acidosis may cause dissociation of transition metals (mainly iron) from binding proteins that catalyze free radical formation. Reactive oxygen species (ROS) can be generated during ATP degradation in the initial phase of ischemia in both the intra- and extracellular space. ROS cause lipid peroxidation of cell membranes, damage of lysosomal membranes, and activation of epithelial cells [9,10].

## 3. Antioxidants with a Potential Nephroprotective Effect

Existing standards in organ procurement and transplantation recommend storing grafts at a reduced temperature using a preservation solution. We have discussed preservative fluids in detail in our previous articles [11,12,13]. Currently, two methods are most commonly used to store organs: by simple hypothermia (the so-called simple cold storage—SCS) and continuous perfusion in hypothermia by vessels using a special pump—HMP (hypothermic machine perfusion) [14]. The first method consists in rinsing the blood off the veins with a cold preservation solution (temperature of 4 °C), which prevents the blood from clotting in the organ and slows down metabolism within the cell, tissue, and organ. Organ cooling reduces the degradation of tissues, reduces the rate of enzymatic reactions, and slows down the consumption of ATP resources [15]. The method of continuous pulse perfusion in hypothermia requires the use of a perfusion device in which the pump pumps the cold preservation solution (4 °C) through the vascular system of the organ in a continuous manner until the time of transplantation. The cooled solution is pumped through the organ’s artery, and after flowing through the organ, it flows out through the vein. This cycle is repeated in the closed pumping system. This method enables regulation of the energy metabolism of the organ and facilitates the elimination of toxins. It also allows for interference in the innate immune response of cells [16]. The conducted research confirms that the use of HMP reduces the frequency of delayed graft function (which minimizes the need for dialysis), shortens the patient’s stay in hospital, and improves graft survival [17]. Currently, portable systems are available on the market that enable kidney transport under conditions of continuous perfusion in hypothermia (Organ Recovery Systems—LifePort). The device allows for the analysis of hemodynamic (flow, resistance) and biochemical parameters of the organ. The time of storing the kidney in the container is up to 72 h [16,18]. There is advanced research on the possibility of storing kidneys and liver by HMP and by normothermia at 37 °C (NMP—normothermic machine perfusion). Both HMP and NMP methods have numerous advantages and disadvantages. Organ storage by normothermia would reduce organ damage resulting from ischemia during hypothermia. Maintenance of the organ under physiological conditions would also allow a determination of the vital functions of the graft and extend the time of its safe storage. The disadvantage of this method is the need to continuously supply oxygen/oxygen carriers during organ storage. The time of cold ischemia should be as short as possible, which should limit the deteriorating organ functions. Hosgood et al. perfused 22 kidneys that were not qualified for transplantation using NMP. They found that the functions of ex vivo grafts were restored and that they were suitable for transplantation [19].

Solutions for organ perfusion and preservation allow for safe storage of organs for a limited period of time. In the case of marginal organs, they show limited effectiveness in maintaining their vital functions. That is why more and more studies are dedicated to modifying the composition of solutions that are to ensure the effectiveness and safety of use as well as the optimal storage time of grafts.

Table 1 and Table 2 list the antioxidants proposed over the past 30 years by researchers to modify the composition of renal preservative solutions.

### 3.1. Potential Mechanisms of Antioxidant Action

The action of antioxidants during ischemia–reperfusion may proceed in three directions. Antioxidants may inhibit oxidation reactions by scavenging reactive oxygen species and oxygen free radicals. These compounds can inhibit the release/action of transition metals that catalyze the Fenton and Haber–Weiss reactions. Antioxidants can activate protein phosphorylation reactions and activate transcription factors that encode the synthesis of enzymatic and non-enzymatic antioxidants. Determining the mechanisms of antioxidant action faces many difficulties due to the complexity of the multifaceted sources of oxidative stress and their dynamic interrelationships [75]. During oxidative stress, ROS are generated in various kidney cells (endothelial, mesangial, and tubular cells) and in the intracellular and extracellular environment, which hinders the therapeutic direction of antioxidants. On the other hand, complete inhibition of ROS signaling could disrupt mechanisms for maintaining cellular homeostasis. For example, complete removal of hydrogen peroxide would impair hemodynamic function [75]. Mitochondria have been identified as a key source and target of excessive ROS production. Currently, many studies are being conducted to develop a therapeutic strategy targeting mitochondria (e.g., a derivative of CoQ10, termed MitoQ, and α-tocopherol-conjugated triphenylphosphonium). The ability of the mitochondrion to build H^+^ transmembrane potential for supporting ATP synthesis is used for this purpose. The hydrophobic part of the compound carrying the positive charge, delocalized on conjugated double bonds, enables the introduction of the antioxidant into the mitochondria [76]. Polyphenolic compounds (e.g., resveratrol, quercetin) show the ability to scavenge ROS, up-regulate Nrf-2 (nuclear factor erythroid 2-related factor 2) signaling, and prevent the release of pro-inflammatory HMGB1 (high mobility group box protein 1) in the circulation [77,78]. The antioxidant properties of bioelements are primarily determined by their role as a component of the active center of antioxidant enzymes (e.g., selenium-dependent glutathione peroxidase, and copper and zinc superoxide dismutase).

### 3.2. Bioelements: Selenium and Zinc

Selenium and zinc influence the maintenance of the correct oxidoreduction balance of the organism. Proper supplementation with these elements significantly influences the correct course of treatment of many chronic metabolic diseases. Selenium is a component of two key amino acids (selenomethionine and selenocysteine) and antioxidant systems with catalase (CAT), superoxide dismutase (SOD), glutathione (GSH), vitamin E, carotenoids, and ascorbic acid. However, the choice of effective selenium supplementation is a complex problem. It results from the narrow therapeutic range of Se (small difference between therapeutic and toxic doses) and the dependence of its action on the chemical form used. A daily selenium intake of 55–70 μg is recommended. This amount is necessary for maintaining optimal glutathione peroxidase (GPX) activity in the body [79]. Selenoproteins are involved in oxidative stress reactions in all cell types. This group of proteins includes glutathione peroxidases (catalyze reduction reactions of hydrogen peroxide and organic peroxides), iodothyronine deiodinases (involved in thyroid hormone metabolism), and thioredoxin reductases (protect cells from oxidative stress) [80]. Selenium has been suggested to potentially affect ROS-dependent pathways [81]. Zinc affects the oxidative-reductive status of cells as a component of superoxide dismutase, which is involved in the neutralization of the superoxide radical. It delays oxidative processes by inducing the expression of metallothioneins acting as free radical scavengers. It shows anti-inflammatory effects and takes part in tissue regeneration [82]. The average zinc requirement is 10–15 mg/day.

Ostróżka-Cieślik et al. [20] conducted a study on the effect of selenium, as a component of Biolasol solution, on the prevention of ischemic damage to nephrons. The study was conducted in a model of isolated porcine kidneys. The fluid was modified with the addition of Se^4+^ (Na_2_SeO_3_) at a dose of 1 µg/L and Se^4+^ system: 1 µg/L + prolactin (PRL): 0.1 µg/L. Selenium ions increased the activity/concentration of the biochemical parameters analyzed: aspartate aminotransferase, alanine aminotransferase, urea, and protein. Optimal renal protection was the modification of the Biolasol fluid composition with the addition of selenite (SO_3_^2−^) and prolactin. The authors suggest that the PRL contained in Biolasol fluid affects the increase in cellular GSH concentration, which is required for the reduction of Se^4+^ to Se^2−^ (selenide). The proposed system: Se + PRL influenced the maintenance of the integrity of the mitochondrial and cytoplasmic membranes of the cells. Lower protein and urea concentrations (markers of renal dysfunction) were also observed. Treska et al. evaluated the effect of selenium addition to HTK fluid on the kidney efficiency of isolated male piglets (the chemical form of selenium was not reported). They confirmed the effect of selenium in minimizing ischemia–reperfusion injury. The concentration of malondialdehyde decreased, with a concomitant increase in the antioxidation capacity of plasma [48,49]. Ostróżka-Cieślik et al. [21] also conducted studies on the effect of zinc on the prevention of ischemic damage in isolated porcine kidneys. They modified Biolasol liquid with the addition of Zn at a dose of 1 µg/L and Zn: 1 µg/L with prolactin (PRL): 0.1 µg/L. Zinc was added in the form of zinc acetate (Zn(CH_3_COO)_2_). An increase in the activity of biochemical markers (ALT, AST, LDH) and the occurrence of hypernatremia were observed. Zinc added to Biolasol + PRL liquid acted as a prolactin inhibitor. Singh et al. [50] studied the effects of University of Wisconsin (UW) fluid modified with the addition of zinc-N-acetylcysteine chelate (0.3–30 mM ZnNAC) on kidneys, in an NRK-52E cell model. They found that ZnNAC (max. effect 1–10 mM) is a potent antioxidant and DNase I endonuclease inhibitor. Its presence in UW fluid formulation decreased caspase-3 activity and the expression of EndoG (endonuclease G).

### 3.3. Vitamin C

Vitamin C (ascorbic acid) is a hydrophilic compound, located in the cytoplasm. It is a strong extracellular and intracellular antioxidant, participating in a number of metabolic processes. It contributes to maintaining the redox balance of the cell by participating in the reduction in superoxide anion radical and hydroxyl radical production, protects against lipid peroxidation and development of inflammation in organs, and supports the immune system by stimulating the production of T lymphocytes and natural killer cells [83]. Together with coenzyme Q and glutathione, it protects mitochondria from oxidative stress. It shows beneficial effects on endothelium (scavenges superoxide anion radical, releases NO from S-nitrosothiols, and stimulates the increased synthesis of citrulline in endothelial cells). It participates in the reconstruction of α-tocopherol and β-carotene from their radical forms [84,85]. It has been suggested that it affects DNA repair by regulating the activity of repair enzymes [86]. In high concentrations, vitamin C can act as a pro-oxidant, promoting oxidation reactions of copper and iron. This can lead to the formation of superoxide and hydroxyl radicals [87]. It is an important antioxidant of extracellular fluids and an important intracellular antioxidant [88,89].

Ostróżka-Cieślik et al. [51] analyzed the effect of ascorbic acid (added to Biolasol solution) on the storage efficiency of isolated pig kidneys. They evaluated the effectiveness of the fluid on the basis of the analysis of ALT (alanine aminotransferase), AST (aspartate aminotransferase), and LDH (lactate dehydrogenase) activities and lactate concentration determined in perfusates taken after 2 h and 48 h of graft storage. The determined ALT, AST, LDH, and lactate activities were within normal limits. Ascorbic acid (dose: 0.088 g/L) had a positive effect on the integrity of the graft cytoskeleton. It reduced ROS formation during renal perfusion and storage and had a protective effect on cell integrity. Vitamin C showed a synergistic effect with prolactin. Norio et al. [72] perfused kidneys with Euro-Collins solution and Euro-Collins solution with added vitamin C immediately before implantation into the recipient. The incidence of delayed graft function (DGF) was similar in both groups (32%/EC + vit. C vs. 29%/EC). A clinical study did not confirm the beneficial effect of vitamin C (dose: 0.5 mg/mL) in kidney transplantation.

### 3.4. Vitamin E

Vitamin E is a hydrophobic compound, located in the cell membrane. Its predominant form in tissues is α-tocopherol [90]. It shows the ability to inhibit lipid peroxidation, thus protecting mitochondrial membranes from oxidative damage. Its antioxidant capacity is mainly due to its ability to deactivate oxygen singlet by quenching. It is also an inhibitor of the lipid peroxidation chain reaction, leading to the formation of the α-tocopheroxyl radical (α-TO^•^) [91]. This radical can be reduced back to its active form by ascorbate. It is suggested that the combination of vitamins C and E enhances the antioxidant effect [92]. Trolox (6-hydroxy-2,5,7,8-tetra-methylchroman-2-carboxylic acid) is a synthetic, hydrophilic analogue of vitamin E. It exhibits the ability to penetrate biological membranes. It protects cells from oxidative damage [93].

McAnulty et al. [52] performed a study to determine whether adding Trolox or ascorbate resulted in a functional benefit to mitochondria of isolated canine kidneys. Kidneys were stored in UW fluid for 48 h in hypothermia. Cortical homogenates were incubated aerobically for 60 min at 37 °C. The authors assessed lipid peroxidation by measuring conjugated dienes, lipid peroxides, and Schiff base. Trolox provided significantly greater antioxidant capacity vs. UW solution (significantly decreased rates of a conjugated diene, lipid peroxide, and Schiff base formation). The addition of ascorbate in turn resulted in increased lipid peroxidation (significantly increased rates of a conjugated diene, lipid peroxide, and Schiff base formation). Trolox slightly inhibited the increase in ascorbate-induced oxidative damage. The authors made an interesting hypothesis that Trolox may be a beneficial component of UW fluid in suboptimal donors (over 70 years of age). The same authors [53] repeated the study in the rabbit kidney cortex slices model. They found that the addition of deferoxamine or Trolox to the storage solution substantially reduced lipid peroxidation. Ascorbate, either alone or combined with either deferoxamine or Trolox, showed pro-oxidative properties. Demirbaş et al. [54] performed a study on the effect of α-tocopherol added to Euro-Collins fluid in a canine kidney autotransplantation model. They found an improvement in creatinine clearance. The rate of lipid peroxidation decreased 24 h after reperfusion.

### 3.5. Carnitine

Carnitine is a vitamin-like substance or “quasi-vitamin”. It has the ability to counteract oxidative stress. It plays an important role in energy production. It participates in the transfer of long-chain fatty acids from the cytosol into the mitochondrial matrix, where beta-oxidation takes place [94,95], regulates nitric oxide levels, increases the activity of enzymes involved in antioxidant defense (glutathione peroxidase, catalase, and superoxide dismutase) [96], and reacts with superoxide and hydrogen peroxide. It shows the ability to chelate iron ions. L-carnitine can stabilize membranes. It prevents lipid peroxidation and DNA cleavage [97,98].

Aslaner et al. [55] investigated the efficacy of carnitine in a Wistar albino rats model. Ringer lactate and UW fluids were modified with the addition of carnitine at a dose of 22 mg/mL. Kidneys were perfused with the preservation solutions through the aorta continuously. Carnitine slowed the increase in LDH levels. The level of malondialdehyde in the tissues decreased. Histopathological examination showed a significant reduction in damage. Mister et al. [56] analyzed the efficacy of propionyl-L-carnitine in an ex vivo rat model of isolated perfused kidney preparation and in vivo in a model of syngeneic kidney transplantation. They added propionyl-L-carnitine at a dose of 1.2 mg/L to the Belzer UW solution (doses of 3.6 and 1.8 mg/mL were toxic). The authors confirmed that the antioxidant was effective in maintaining normal renal function during ischemia–reperfusion. The amount of released LDH decreased in the kidney group washed with modified fluid (but not normalized). Histological changes in the kidneys were significantly less compared with the control group. The structure of the tubular cells did not change. The authors suggest that propionyl-L-carnitine protects against oxygen free radical generation and decreases lipid peroxidation.

### 3.6. Flavonoids

Flavonoids are plant-derived compounds composed of two aromatic rings joined by a pyran or pyrone ring [99]. The main flavonoid classes include flavones (e.g., luteolin), flavanols, flavonols (e.g., kaempferol, quercetin, fisetin), flavanones, and anthocyanidins. They exhibit antioxidant activity by several mechanisms. They prevent the formation of reactive oxygen species (ROS) by inhibiting the activity of enzymes involved in their production. They act as chelating agents for transition metal ions (mainly iron), reducing the concentration of ROS. They have the ability to capture free oxygen radicals (superoxide anion radical, superoxide radical, hydroxyl radical) and to quench singlet oxygen. They inhibit lipid peroxidation processes by interrupting cascades of free radical reactions [100,101,102]. They have been suggested to be effective in the prevention and therapy of acute kidney injury (AKI) and chronic kidney disease (CKD) [103].

Ahlenstiel et al. [57] performed analyses to determine the renoprotective properties of bioflavonoids. They conducted their study on a cell model LLC-PK1 (a proximal tubular epithelial cell line of pig origin), which they stored in the University of Wisconsin (UW) or Euro-Collins (EC) solution in hypothermia for 20 h. They found that bioflavonoids minimized renal tubular cell injury. They demonstrated efficacy for luteolin, quercetin, kempferol, myricetin, morin, catechin, silibinin (100 μmol/L each), and fisetin (50 μmol/L). Bioflavonoids protected against structural (LDH release 0–2.4%) and functional (MTT test 103–110%) cell damage. MDA release decreased by 75–85% in UW solution modified with quercetin, kaempferol, and luteolin and by 85–90% in EC solution modified with quercetin, kaempferol, and luteolin. The flavonoids (and their optimal doses) that showed the highest efficacy are listed in Table 2. Apigenin, naringenin, and rutin did not show cytoprotection. The authors suggest that the renoprotective effect of flavonoids depends on the number of hydroxyl-substituents and differences in lipophilicity. Gochi et al. [58] found effective effects of quercetin (dose: 33.1 μM) and sucrose added to UW fluid during cold storage (CS) and hypothermic oxygenated perfusion (HOPE) in a porcine model of renal autologous transplantation and in a BHK-21 cells (baby hamster kidney fibroblast cells) model. Quercetin improved the survival of BHK-21. The LPO level was significantly lower on the second day after kidney transplantation. The creatinine level was also significantly lower. The modified fluid minimized I/R damage and improved renal function. The addition of 10 μM quercetin to UW solution before cold storage prevented most of the morphological changes in the kidney. Quercetin decreased ROS generation by 80% [60]. The authors suggest the addition of quercetin to the solution preservation can improve kidney preservation and could potentially enhance the outcomes of kidney transplantation.

### 3.7. Resveratrol

Resveratrol (3,4′,5-trihydroxy-trans-stilbene, RSV) is a natural polyphenol that belongs to the class of stilbenes [104]. Stilbenes are a group of polyphenols composed of two phenolic rings, connected by a two-carbon methylene bridge. RSV exhibits lipophilic characteristics. It is a strong antioxidant that scavenges mitochondrial reactive oxygen species and diminishes quinone-reductase-2 activity. It may increase the activity of superoxide dismutase (SOD) and glutathione peroxidase (GPx), enhance the potential of mitochondrial membrane and ATP levels, and decrease the level of malondialdehyde (MDA) [105,106]. Its action is related to the modulation of many cellular processes. It increases the activity of the adenosine monophosphate (MP)-activated protein kinase (AMPK). AMPK can phosphorylate and activate PGC1α (peroxisome proliferator-activated receptor-γ coactivator-1α), which promotes mitochondrial biogenesis [107]. It is a potential tool for regulating SIRTs (sirtuins), histone deacetylases regulatory enzymes of genetic materials. Resveratrol can reduce inflammation and oxidative stress by activating Nrf2 (nuclear factor (erythroid-derived 2)-like 2) and SIRT1 (silent information regulator T1) signaling. It reduces the stimulation of the NF-κB (nuclear factor κB) and the release of endogenous cytokines [108,109]. 

Soussi et al. [59] in their study used Vectisol^®^ (resveratrol–cyclodextrin conjugate) containing 2.2 mg trans-resveratrol. They conducted the research in a preclinical pig model of kidney autotransplantation. They flushed the kidneys with the kidney perfusion solution (Celsior or UW or HTK or SCOT15) or kidney perfusion solution modified by Vectisol^®^. Resveratrol had a positive effect on histological structure and graft function and slowed interstitial fibrosis and tubular atrophy at one 1-month post-transplantation. The substance prevented delayed graft function after 24 h of cold ischemic time. The authors observed a decrease in oxidative stress and decreased levels of apoptosis. Supplementation of resveratrol improved glomerular filtration as well as decreasing proteinuria and the early levels of circulating SOD (superoxide dismutase) and ASAT (asparagine aminotransferase). Karhumäki et al. [60] studied the efficacy of resveratrol in a renal tubular epithelial cell (LLC-PK1) model. UW fluid was modified with RSV (dose: 1 μM or 10 μM). Resveratrol (dose: 1 μM) effectively prevented LDH release but was less effective in protecting against ATP depletion. RSV decreased ROS generation by 60% and reduced oxidative stress.

### 3.8. Tanshinone IIA

Tanshinone IIA (TIIA) is a lipophilic diterpene extracted from the root of Salvia miltiorrhiza. It exhibits anti-inflammatory activity and prevents oxidative stress and apoptosis. It decreases the levels of malondialdehyde (MDA) and oxidized low-density lipoprotein (oxLDL) and increases the activity of glutathione peroxidase (GPx), superoxide dismutase (SOD), and catalase [110]. It decreases H_2_O_2_ levels and reduces the accumulation of reactive oxygen species (ROS) [111]. It stabilizes vascular endothelial function and inhibits expression of inflammatory cytokines IL-1β and TNF-α [112]. Zhang et al. [61] analyzed the effect of Tanshinone IIA on the storage efficiency of isolated rat kidneys. They added the antioxidant to Celsior fluid at a dose of 100 µmol/L. They found an increase in superoxide dismutase (SOD) activity and a decrease in malonaldehyde (MDA) in kidneys stored in modified fluid (Celsior + TIIA) at 24 and 48 h. CHOP (C/EBP homologous protein) expression and caspase-12 mRNA levels were also lower in this group. The authors suggest that TIIA may improve long-term renal preservation.

### 3.9. Lecithinized Superoxide Dismutase (Lec-SOD)

Lecithinized superoxide dismutase (lec-SOD; lecithinized human copper/zinc SOD) is a modified form of SOD showing high clinical efficacy. SOD therapy is not very effective due to its short half-life (approximately 6 min), low stability in plasma, and low affinity for the cell membrane. Lec-SOD shows high affinity with cell membranes, high stability in plasma, and higher pharmacological activity [113,114,115]. It exerts antioxidant and anti-apoptotic effects. By scavenging free oxygen radicals, it protects against the toxicity of reactive oxygen species [116]. It inhibits the increases in the expression of markers of fibrosis α-smooth muscle actin and collagen [117]. Nakagawa et al. [62] investigated the efficacy of Lec-SOD in a rat kidney allograft model. Grafts were stored in Marshall’s and Marshall’s+ Lec-SOD solution (50 μg/mL dose). Lec-SOD minimized ischemia–reperfusion damage. It inhibited the early inflammatory response (but not long-term alloimmune response). Apoptosis levels were lower in lec-SOD allografts 24 weeks after surgery. Leukocyte infiltration was significantly lower at day 1 after 18 h of cold preservation in the Marshall’s + Lec-SOD group. There was a protective effect of antioxidant on renal endothelial cells after 18 h of cold preservation.

### 3.10. Mitoquinone

Mitoquinone (MitoQ; 10-(4,5-dimethoxy-2-methyl-3,6-dioxo-1,4-cyclohexadien-1-yl)decyl triphenylphosphonium) is a synthetic derivative of coenzyme Q10. It is a potent therapeutic antioxidant with a ubiquinone moiety attached to a lipophilic triphenylphosphonium/TPP+ cation. It belongs to the family of mitochondria-targeted antioxidants (>90% of the compound is bound to the mitochondrial membrane) [118]. MitoQ reduces mitochondrial oxidative stress and inhibits lipid peroxidation by blocking hydroxyl radical attack. It is characterized by high bioavailability. Currently, the FDA has not yet approved MitoQ as a drug [119,120]. Mitchell et al. [63] placed renal cells or isolated rat kidneys in UW solution modified with the addition of mitoquinone (dose: 1 μM in vitro; 100 μM ex vivo). MitoQ treatment decreased mitochondrial superoxide generation production ~2-fold. Mitoquinone prevented mitochondrial damage and improved renal morphology. The renoprotective effect of MitoQ was confirmed in a further study. Parajuli et al. [64] flushed the kidneys with cold Belzer’s solution with mitoquinone at a dose of 100 μM. MitoQ improved mitochondrial function and reduced oxidative stress in the renal tubules after 24 and 48 h. There was less accumulation of nitrotyrosine protein in the kidney cortical and decreased mitochondrial superoxide production, including hydrogen peroxide and peroxynitrite. Mitoquinone blocked renal cell death after 48 h. TUNEL-positive signals were detected in the kidney.

### 3.11. Edaravone

Edaravone (3-methyl-1-phenyl-2-pyrazolin-5-one, MCI-186, Radicut^®^) is a synthetic, potent free radical scavenger. It was developed in Japan by Mitsubishi Tanabe Pharma Corporation and introduced into therapeutics in 2001. Radicut^®^ is a drug used in therapy to treat acute ischemic stroke (AIS) patients [121]. In 2017, it was approved by the U.S. Food and Drug Administration (FDA) for the treatment of ALS (amyotrophic lateral sclerosis) [122]. Edaravone shows the ability to quench hydroxyl radical and inhibit hydroxyl radical-dependent and hydroxyl radical-independent lipid peroxidation. It inhibits peroxyl radical-induced peroxidation systems. It is suggested that its effectiveness corresponds to the action of the system: vitamin C with vitamin E [123]. Edaravone prevents mitochondrial oxidative stress [124] and protects endothelial cells against damage by reactive oxygen species [125]. Edaravone reduced cold I/R injury in a canine kidney autotransplantation model [65]. Kidneys were stored 72 h in cold HTK fluid modified with edaravone at a dose of 50 μM. It was additionally applied at harvest and at reperfusion (3 mg/kg). Edaravone suppressed lipid peroxidation in renal tubular cells and ameliorated kidney dysfunction. The agent inhibited P-selectin expression in renal endothelial cells, improved renal vascular resistance, and maintained renal tissue blood flow. Edaravone increased urine production and Cr clearance. The agent lowered the mean serum creatinine.

### 3.12. Nicaraven

Nicaraven (N,N′-propylenebisnicotinamide; AVS) is an antioxidant that exhibits hydroxyl radical scavenging activity in vitro. The agent has hydrophilic and lipophilic chemical properties. One molecule of nicaraven directly neutralizes two hydroxyl radicals by intramolecular π-dimerization. Nicaraven has the ability to suppress lipid peroxidation [126]. The drug prevents vascular constriction [127]. Lin et al. [128] found that AVS inhibits TNFα-induced endothelial activation and inflammatory. Masaki et al. [66] conducted efficacy studies of nicaraven in two models. In the first, isolated rat kidneys were flushed with UW solution, to which the drug was added at doses of 2.8 mg/dL, 28 mg/dL, and 56 mg/dL. In the second model, isolated canine kidneys were washed with Euro-Collins fluid plus 28 mg/dL AVS. The authors suggest that the drug may have inhibited the generation of lipid peroxides due to hyperoxidation. The nicaraven-treated grafts showed less tubular necrosis compared with the control group. Interestingly, the urine volume in rats whose kidneys were treated with nicaraven was significantly higher than in the control group. This is probably related to the improvement in hypoperfusion of blood flow in the kidney [129].

### 3.13. Propofol

Propofol is a lipophilic antioxidant and a widely used anesthetic. The agent has a chemical structure that is similar to alpha-tocopherol (which also contains a phenolic OH-group). It has been suggested that it may prevent lipid peroxidation in cell membranes and that it can be a potent modifier of biomembranes [67,130]. The drug scavenges reactive oxygen species (ROS) to generate less reactive phenoxyl radicals [2]. Snoeijs et al. [67] added a cyclodextrin propofol preparation to a 140 μM water-soluble HTK solution. The modified solution was used for intra-arteriolar flushing of porcine kidneys. Renal exposure to propofol prevented lipid peroxidation and reduced the increase in renovascular resistance at reperfusion after autotransplantation. Propofol improved renal function in the early post-transplant period, but leucocyte infiltration in kidneys was not diminished. The authors suggest that propofol may attenuate hypothermic and ischemic acute renal damage.

### 3.14. Deferoxamine

Deferoxamine (Desferrioxamine, DFO) is a bacterial siderophore produced by Streptomyces pilosus. The drug chelates free iron ions in a 1:1 ratio (forming a ferixamine complex), making it a potential modulator of the oxidative stress involved. Iron catalyzes reactive oxygen species (ROS) directly via the Fenton and Haber–Weiss reaction. The Fenton reaction is between ferrous ion (Fe^2+^) and hydrogen peroxide (H_2_O_2_), which is mainly produced by superoxide dismutase (SOD) [131,132,133]. DFO stabilizes HIF-1α (hypoxia-inducible factor-1α) under normoxic conditions [134].

Huang et al. [68] studied the effectiveness of deferoxamine using a syngeneic kidney transplantation model. The UW fluid was modified by the addition of DFO at 0.125 or 0.625 mM. Deferoxamine significantly increased glomerular filtration rate (GFR), and renal blood flow (RBF). DFO suppressed renal F2-isoprostanes (vasoactive lipid peroxidation products) and apoptotic and necrotic tubular injury (3 days post-transplantation). In another study [69], the same authors found that deferoxamine at a dose of 2.5 mM significantly suppressed the BDI (bleomycin-detectable iron) and LDH (lactic dehydrogenase) release and that it reduced the ultrastructural changes. Salahudeen et al. [70,73,74] conducted a series of studies to test the efficacy of deferoxamine and 2-methyl aminochroman (2-MAC) as a component of UW fluid. Lazaroid antioxidant (2-methyl aminochromane/2-MAC) is a steroid compound that inhibits lipid peroxidation and cell proliferation. 2-MAC is a strong oxygen free radical scavenger. Antioxidants were protective on renal tubular cells. DFO caused significant suppression of vasoconstrictive isoprostane formation. The drug suppressed the 48 h cold-induced LDH release and prevented depletion in glutathione. Deferoxamine lowered the hydrogen peroxide levels and prevented cold-induced ATP reduction. Cell proliferation was significantly higher in the presence of DFO and 2-MAC [70]. The addition of deferoxamine and 2-methyl aminochromane to UW solution inhibited necrotic cell death and preserved mitochondrial structure [73].

### 3.15. PrC-210

PrC-210 aminothiol (3-(methyl-amino)-2-((methylamino)methyl)propane-1-thiol) is a potent antioxidant that can be used intravenously, orally, and topically, showing few side effects [135]. Aminothiol has a small molecular size, which allows for efficient transmembrane diffusion. Amines on an alkyl backbone enable strong ionic interaction with DNA and scavenging of ROS around DNA. PrC-210 scavenges recurrent oxygen species and protects plasmid DNA from ROS-induced damage [136]. It has been observed that the PrC-210 minimizes the risk of cell death in myocardial infarctions in a mouse model after I/R [137]. The antioxidant effect of PrC-210 is dose-dependent. Its ROS scavenging capacity was found to be optimal in the dose range of 0.5 mmol/L–2.3 mmol/L. PRC-210 has the advantage of rapid (within seconds) and long-term antioxidant activity (up to several hours). Its disadvantage is its limited applicability after the damage has occurred. PRC-210 has shown high efficacy in damage prevention [135]. In humans, it has a radioprotective effect against lethal radiation dose [138]. 

Verhoven et al. [47] investigated the efficacy of PrC-210 in suppressing cold-ischemia injury in the Sprague-Dawley rats model. The authors measured kidney-activated caspase-3 level (a marker of cell death) and performed histological analysis. They observed that kidney-activated caspase levels decreased after 30 h of cold-ischemia. PrC-210 conferred 100% protection against ROS damage. In turn, Goesch et al. [71] added PRC-210 to UW fluid and investigated its efficacy in an allograft kidney transplant model. They performed analysis of kidney histology and inflammatory cytokine levels. They found that renal histological damage was reduced, cytokine levels (TIMP-1, TNF-alpha and MIP-3A/CCL20) decreased, and creatinine and BUN levels were low. PrC-210 showed a protective effect in allogeneic transplantation.

### 3.16. Concluding Remarks

The studies cited were carried out in various animal models, with several in human models. Their aim was to obtain information on the short-term efficacy of antioxidants. Some of the studies provide contradictory information on the efficacy of antioxidants (vitamin C, selenium). There have been no long-term studies indicating the possible effect of antioxidants after transplantation. There have also been no studies indicating whether antioxidants acted by feeding the pool of the antioxidant system or whether they showed additive and/or synergistic effects. However, the study of antioxidants has provided valuable multifaceted results regarding their efficacy in protecting the kidney during ischemia, which represents a high potential for informing future clinical trials.

**Figure 1:**
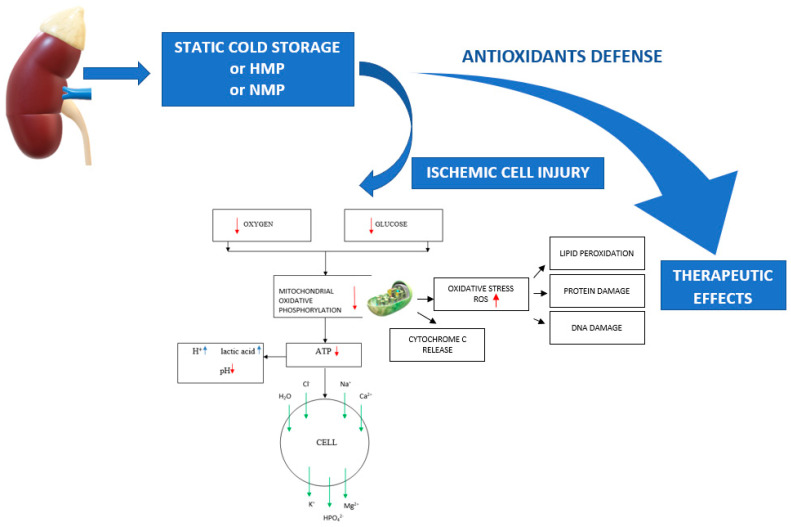
Processes involved in kidney storage, ischemic damage, and antioxidant protection.

Figure 1 shows the processes involved in kidney storage, ischemic damage, and antioxidant protection.

## 4. Clinical Potential of Antioxidants and Future Perspectives

Antioxidants represent a promising group of compounds with which to modify the compositions of organ perfusion and preservation fluids to increase their quality and efficacy. Available preclinical and clinical data mostly indicate their positive effects during kidney preservation before transplantation. Studies have been performed in different animal species but have not been followed up with clinical trials in humans. The selection of markers of modified fluid efficacy is also wide. Further research should concentrate on conducting larger scale clinical trials. It is necessary to determine the optimal dose of antioxidant and the safety of its use (taking into account the direct effect of the antioxidant on the organ). There is also a lack of information on the behavior of these compounds once they enter the cell during kidney flushing. The area in need of development is the technology for preparing preservative fluids containing antioxidants. It is important to determine the stability of fluids modified with antioxidants. The dose and type of antioxidant significantly affect the stability of the preservative fluid and consequently the safety of its use [139,140,141]. It has been shown that the addition of zinc to Biolasol fluid decreases its stability by 30.5%, while the addition of selenium ions increases its stability by 8.21% [139]. It is important to develop procedures for the preparation of a preservative fluid containing an easily oxidizable substance. Recent work has shown that glutathione added to UW fluid undergoes autooxidation during production and that the fluid is contaminated with trace amounts of iron ions (catalyst for the oxidation reaction). This significantly affected the limited effectiveness of the fluid [142]. There is increasing evidence in the literature that combinations of antioxidants may be more effective. Additionally, combining FDA-approved drugs with natural antioxidants may be more effective. The possibility of introducing an antioxidant in the form of a carrier system into the fluid formulation should be considered. Carrier systems are stable during production and storage of the preparation, stable to electrolytes, and release the substances slowly over time, and their effect may continue after transplantation. 

## Figures and Tables

**Table 1:** Antioxidant types and mode of the action.

Antioxidant	Class	Half-Life (t½)	Serum or Plasma Concentrations	Mechanism of Action	Activities	References
Selenium	Mineral	65–115 days	98–108 µg/L	Acts as a cofactor for enzymatic antioxidants	Antioxidant, anti-inflammatory, antimutagenic, anticarcinogenic, antiviral, antibacterial, antifungal	[20]
Zinc	Mineral	16–43 days	11–18 µmol/L	Acts as a cofactor for enzymatic antioxidants	Antioxidant, anti-apoptotic, anti-inflammatory, anti-allergic	[21]
Vitamin C	Vitamin	14–23 days	54–91 µmol/L	Inhibits the oxidation processes by scavenging free radicals	Antioxidant, immunomodulatory, antiviral, anti-inflammatory	[22,23]
Vitamin E	Vitamin	18–81 h	21–27 µmol/L	Inhibits the oxidation processes by scavenging free radicals	Antioxidant, immunomodulatory, anti-inflammatory	[23,24]
L-carnitine	Non-protein amino acid	10–45 h	25–50 µmol/L	Iron chelator; inhibits the oxidation processes by scavenging free radicals and acts as an energy source	Antioxidant, anti-inflammatory, anti-obesity, anti-atherosclerosis, anti-anemia, anticancer, immunomodulatory, regulator of lipid metabolism	[25,26]
Flavonoids	Flavone (luteolin) Flavonols (kaempferol, quercetin, fisetin)	2–28 h	<1 µmol/L	Inhibits the oxidation processes by scavenging free radicals	Antioxidant, anti-inflammatory, anti-allergic, antiviral, antithrombotic, antimutagenic, antineoplastic, hepatoprotective, renoprotective	[27,28]
Resveratrol	Phenol	2–4 h	-	Inhibits the oxidation processes by scavenging free radicals	Antioxidant, anti-inflammatory, anti-apoptosis, antitumor	[29,30]
Tanshinone IIA	Terpenoid; isolated from *Salviae miltiorrhizae*	2–5 h	-	Regulates the levels of antioxidant enzymes	Antioxidant, anti-inflammatory, antibacterial, antiviral, antineoplastic, vasodilator, antithrombotic, anti-atherosclerosis, antiallergic	[31,32,33]
Lec-SOD (lecithinized superoxide dismutase)	Enzyme	1.54 days	-	Inhibits the oxidation processes by scavenging free radicals	Antioxidant, anti-inflammatory, anti-apoptosis	[34,35,36]
MitoQ	Quinone	1.5 days	-	It is reduced by the respiratory chain to its active ubiquinol form, which is antioxidant that prevents lipid peroxidation and mitochondrial damage.	Antioxidant, anti-inflammatory	[37,38]
Edaravone	Pyrazolone	4.5–6 h	-	Inhibits the oxidation processes by scavenging free radicals	Antioxidant, anti-inflammatory, anti-apoptotic, antinecrotic	[39,40,41]
Nicaraven	Pyridine (nicotinamide)	no data available	-	Inhibits the oxidation processes by scavenging free radicals	Antioxidant, anti-inflammatory	[42,43]
Propofol	Phenol	1.5–31 h	-	Inhibits the oxidation processes by scavenging free radicals	Antioxidant, anti-inflammatory	[44,45]
Deferoxamine	Chelating agent	0.5–1 h	-	Iron chelator; controls the production of metal catalyzed free radicals	Antioxidant, angiogenic	[46]
PrC-210	Aminothiol	3.5 h (pH = 7.2)	-	Inhibits the oxidation processes by scavenging free radicals	Antioxidant, radioprotector	[47]

**Table 2:** Strategies based on modifications of preservation solutions for kidney transplantation.

Author, Year of Publication	Antioxidant	Species	Preservation Solution Modification /Cold Ischemia	Outcome Measures, (Intervention, I/Control, C)	Antioxidant Dose	Effects of Antioxidant
**Animals**						
Ostróżka-Cieślik et al., 2020 [20]	Selenium	Pig	Biolasol 2 h, 48 h, 4 °C; SCS	I: Biolasol + Se^4+^and PRL C: Biolasol	Se: 1 µg/L PRL: 0.1 µg/L	↓ ALT, AST, protein, urea Se^4+^ and PRL affects the integrity of mitochondrial and cytoplasmic membranes
Treśka et al., 2003 [48]	Selenium	Piglets	HTK 24 h; 4 °C; SCS	I: HTK + Se C: HTK	Se: 200 µg	↓ MDA Reduced the production of FOR ↑ AOC
Treśka et al., 2003 [49]	Selenium	Piglets	HTK 24 h; 4 °C; SCS	I: HTK + Se C: HTK	Se: 200 µg	↓ MDA Reduced the production of FOR ↑ AOC Decreased the intensity of IRS
Ostróżka-Cieślik et al., 2021 [21]	Zinc	Pig	Biolasol 2 h, 48 h, 4 °C; SCS	I1: Biolasol + Zn^2+^ I1: Biolasol + Zn^2+^ and PRL C: Biolasol	Zn: 1 µg/L PRL: 0.1 µg/L	↓ AST, ALT, and LDH ↑ Na^+^, K^+^ Acted as a prolactin inhibitor
Singh et al., 2013 [50]	ZnNAC	NRK-52E cells	UW 24 h; 0 °C; SCS	I: UW + ZnNAC C: UW	0.3–30 mM max. effect: 1–10 mM	Decreased DNA fragmentation Decreased the amount of active caspase-3 Decreased the expression and nuclear import of EndoG
Ostróżka-Cieślik et al., 2018 [51]	Vitamin C	Pig	Biolasol 2 h, 48 h, 4 °C; SCS	I: Biolasol + Vit.C C: Biolasol	0.088 g/L	↓ ALT, AST, LDH Reduced oxidative stress
McAnulty et al., 1996 [52]	Vitamin C Trolox	Dog kidney	UW 48 h; 2 °C; SCS	I1: UW+ Vit.C I2: UW + Trolox C: UW	Ascorbic acid: 1 mM Trolox: 200 μM	Reduced oxidative stress ↓ Lipid peroxidation
McAnulty et al., 1997 [53]	Vitamin C Trolox Deferoxamine	Rabbit kidney cortex slices	UW 18 h; 5 °C; SCS	I1: UW + Vit.C I2: UW +Trolox I3: UW + deferoxamine C: UW	Ascorbate: 1 mM Trolox: 1 mM DFO: 1 mM	Reduced oxidative stress ↓ Lipid peroxidation Ascorbate was prooxidant when combined with deferoxamine or Trolox
Demirbaş et al., 1993 [54]	α- tocopherol	Dog	EC 24 h; 4 °C; SCS	I: EC + α-tocopherol C: EC	30 mM/L	↓ Lipid peroxidation
Aslaner et al., 2018 [55]	L-carnitine	Wistar albino rat	Ringer lactate, UW 24 h, 48 h, 72 h; 4 °C	I1: UW + l-carnitine I2: Ringer lactate + l-carnitine C1: UW C2: Ringer lactate	22 mg/mL	↓ MDA ↓ LDH
Mister et al., 2002 [56]	Propionyl-L-carnitine	Rat	UW 4 h, 4 °C; SCS	I: UW + propionyl-L-carnitine C: UW	1.2 mg/mL	↓ LDH Prevents polymorphonuclear cell graft infiltration Reduces tubular injury
Ahlenstiel et al., 2006 [57]	Bioflavonoids	LLC-PK1 cells	UW 20 h; 4 °C; SCS EC 20 h; 4 °C; SCS	I1: UW + luteolin I2: UW + quercetin C1: UW I1: EC + luteolin I2: EC + quercetin C2: EC	Luteolin (in UW): 12.5–50 µM Quercetin (in UW): 25 µM Luteolin (in EC): >50 µM Quercetin (in EC): >50 µM	↓ LDH ↓ MDA Protection of renal proximal tubular
Gochi et al., 2020 [58]	Quercetin	BHK-21 cells or Pig	UW 72 h, 4 °C; SCS 24 h, 4 °C; SCS 22 h, 4 °C; SCS/MP; 2 h HOPE	I: UW + quercetin + sucrose C: UW	Quercetin: 33.1 µM Sucrose: 0.1 M	Increased cell viability ↓ Lipid peroxidation ↓ creatinine Reduced oxidative stress ↓ I/R injury
Soussi et al., 2019 [59]	Resveratrol	Pig	KPS (Celsior or UW or HTK or SCOT15) 24 h; 4 °C/CS; HMP	I: KPS + Vectisol^®^ C1: KPS C2: KPS + cyclodextrins	Vectisol^®^ (2.2 mg trans-resveratrol and 1577.8 mg cyclodextrins)	Slow-down of the loss of renal functions Decrease in apoptosis Reduced oxidative stress
Karhumäki et al., 2007 [60]	Resveratrol quercetin, epigallocatechin gallate (EGCG), butylated hydroxyanisol (BHA)	LLC-PK1 cells	UW 16–18 h, 5 °C	I1: UW + resveratrol I2: UW + quercetin I3: UW + EGCG I4: UW + BHA C: UW	0.1–30 μM	Reduced oxidative stress Prevented most of the morphological changes (EGCG had no effect) ↓ LDH
Zhang et al., 2012 [61]	Tanshinone IIA	Sprague-Dawley (SD) male rat	Celsior 24 h, 48 h; 4 °C; SCS	I: Celsior + Tanshinone IIA C: Celsior	100 µM/L	↓ MDA ↑ SOD ↓expression of CHOP and caspase-12
Nakagawa et al., 2002 [62]	Lec-SOD	Fisher rat	Marshall’s solution 1 h, 18 h; 4 °C; SCS	I: Marshall’s solution + lec-SOD C: Marshall’s solution	50 μg/mL	↓ proteinuria ↓ inflammatory response involving granulocytes and macrophages ↓ apoptotic cells ↑ expression of intracellular adhesion molecule-1 (ICAM-1)
Mitchell et al., 2011 [63]	MitoQ	Renal cells or Rat	UW 4 h; 4 °C; SCS	I: UW + MitoQ C1: UW C2: UW + DecylTPP	MitoQ: 1 μM in vitro or 100 μM ex vivo DecylTPP: 100 μM	Prevented mitochondrial dysfunction Improved cell viability Improved renal morphology
Parajuli et al., 2012 [64]	MitoQ	Pig	Belzer’s solution 24 h, 48 h; 4 °C; SCS	I: Belzer’s solution + MitoQ C: Belzer’s solution	100 µM	Improved complex II/III respiration of the electron transport chain Reduced oxidative stress ↓ tubular damage improving mitochondrial function
Tahara et al., 2005 [65]	Edaravone	Dog	HTK 72 h; 4 °C; SCS	I: HTK + edaravone C: HTK	50 μM	↑ urine output ↑ glomerular filtration rate ↓ serum creatinine ↓ renal vascular resistance improved tubular cell function
Masaki et al., 1998 [66]	Nicaraven	Rat, dog	UW 48 h; 4 °C; SCS EC 72 h; 3 °C; SCS	I1: UW + nicaraven C1: UW I2: EC + nicaraven C2:EC	2.8 mg/dL; 28 mg/dL; 56 mg/dL 28 mg/dL	↓ tubular necrosis Well-preserved mitochondrial cristae ↓ Lipid peroxidation
Snoeijs et al., 2011 [67]	Propofol	Male pig	HTK 22 h; 4 °C; SCS/HMP	I: HTK + propofol C: HTK	140 μM	Preventing lipid peroxidation ↓ MDA
Huang et al., 2003 [68]	Deferoxamine	Wistar Furth rat	UW 18 h; 4 °C; SCS	I: UW + deferoxamine C: UW	0.125 mM; 0.625 mM	↑ glomerular filtration rate (GFR) ↑ renal blood flow (RBF) ↓ renal F2-isoprostanes (vasoactive lipid peroxidation products)
Huang et al., 2002 [69]	Deferoxamine	Rat	UW 48 h; 4 °C; SCS	I: UW + deferoxamine C: UW	2.5 mM	↓ BDI, LDH Mitochondrial swelling and cell Injury were markedly suppressed
Salahudeen et al., 1999 [70]	Deferoxamine	LLC-PK1 Rat	UW 48 h; 4 °C; SCS	I: UW + deferoxamine C: UW	DFO: 1 mM or 1 μM	↓ F2-isoprostane formation
Verhoven et al., 2020 [47]	PrC-210	Rat	UW 48 h; 4 °C; CS	I: UW + PrC-210 C: UW	0–40 mM/L	↓ caspase-3 reduced renal tubular injury
Goesch et al., 2021 [71]	PrC-210	Rat	UW 15 sec, 15–25 °C, perfusion in situ	I: UW + PrC-210 C: UW	30 mM	Histologic damage and mononuclear infiltration were reduced ↓ creatinine ↓ BUN activated caspase and cytokine were reduced
**Humans**						
Norio et al., 2003 [72]	Vitamin C	Human	EC 4 °C; SCS	I: EC + Vitamin C C: EC	0.5 mg/mL	No advantage
Salahudeen et al., 2000 [73]	Deferoxamine 2-methyl aminochroman	Human renal tubular cell	UW 48 h; 4 °C; SCS	I: UW + deferoxamine I2: UW + 2-methyl aminochroman C: UW	DFO: 0.25 mM or 2.50 mM 2-MAC: 1.56 μM	↓ LDH ↑ proliferation rate structural protection of the cells
Salahudeen et al., 2001 [74]	Deferoxamine 2-methyl aminochroman	Human renal proximal tubular cells	UW 12 h, 24 h, 36 h, 48 h; 4 °C; SCS	I: UW + deferoxamine I2: UW + 2-methyl aminochroman C: DMEM (37 °C)	DFO: 2.50 mM 2-MAC: 1.56 μM	↓ necrotic cell death ↓ apoptotic cell death: 2-MAC: 3.1%; DFO: 3.2%.

Abbreviations: ALT, alanine transaminase; AST, aspartate transaminase; PRL, prolactin; MDA, malondialdehyde; HTK, histidine–tryptophan–ketoglutarate solution; SCS, simple cold storage; HMP, hypothermic machine perfusion; FOR: free oxygen radicals; AOC, antioxidation capacity of plasma; IRS, ischemia–reperfusion syndrome; LLC-PK1 cells, a proximal tubular epithelial cell line of pig origin; UW; University of Wisconsin; EC, Euro-Collins; LDH, lactate dehydrogenase; ZnNAC, zinc-N-acetylcysteine; NRK-52E cells, normal rat tubular epithelial NRK-52E cells; EndoG, endonuclease G; DMEM, Dulbecco’s modified Eagles medium; LLC-PK1, renal tubular epithelial cells; CHOP, C/EBP homologous protein; TBARS—thiobarbituric acid reactive substance; HOPE, hypothermic oxygenated perfusion; KPS, kidney perfusion solution; BHK-21 cells, baby hamster kidney fibroblast cells; DecylTPP, decyl(triphenyl)phosphonium; BDI, bleomycin-detectable iron; BUN, blood urea nitrogen; ↑ increase ↓ decrease.

## Data Availability

The data presented in this study are available on request from the corresponding author.
